# The monogenic forms of obesity in Saudi families

**DOI:** 10.1093/hmg/ddag054

**Published:** 2026-10-19

**Authors:** Sultanah Turki Almolafikh, Ali A Alshatwi, Razan Orfali, Haya AlSaud

**Affiliations:** Department of Food Science and Nutrition, College of Food and Agricultural Sciences, King Saud University,, P.O. Box 2460, Riyadh 11451, Saudi Arabia; Department of Food Science and Nutrition, College of Food and Agricultural Sciences, King Saud University,, P.O. Box 2460, Riyadh 11451, Saudi Arabia; Department of Pharmacology, College of Medicine, Imam Mohammad Ibn Saud Islamic University (IMSIU), Riyadh 13317, Saudi Arabia; King Faisal Specialist Hospital Research Centre, Riyadh, 13458, Saudi Arabia; King Abdulaziz City for Science and Technology, Riyadh 12354, Saudi Arabia; Hevolution Foundation, King Saud University, Riyadh 13519, Saudi Arabia

**Keywords:** consanguineous families, syndromic of obesity, monogenic, rare disease, obesity, heterozygosity, homozygosity

## Abstract

**Background:**

Obesity is a multifactorial disorder with a substantial yet incompletely defined genetic component. Rare monogenic variants, particularly in consanguineous populations, may account for part of the ‘missing heritability.’

**Objectives:**

To delineate the genetic contribution to severe obesity in individuals from Saudi Arabia by pinpointing known and novel monogenic variants in families presenting with extreme phenotypes.

**Methods:**

Eight multiplex Saudi families (each comprising 9 members; range: lean body weight to severe obesity) were enrolled. Adults had a BMI of ≥30 kg/m^2^; children exceeded the 90th percentile (overweight) or the 97th percentile (obese). Whole-exome sequencing (WES) was performed on samples collected from two children in each family, including one child with obesity and one control child without obesity. Variant calling files were filtered for quality, frequency < 1%, and predicted functional impact. Candidate loci were prioritized using Moon (Diploid) software and manual curation, then validated by Sanger sequencing.

**Results:**

Initial filtering revealed 40 rare, potentially pathogenic variants in 35 genes shared among affected relatives. Prioritization highlighted 28 variants in 19 candidate genes, encompassing established obesity-related genes (e.g. *LEPR, MC4R, BDNF*) and putative novel loci linked to appetite regulation, lipid metabolism, and energy balance. Segregation analysis identified both heterozygous and homozygous variants across affected individuals, reflecting variability in inheritance patterns. Seven previously reported candidate genes did not show pathogenic variants in this cohort. Carriers of prioritized variants exhibited significantly higher BMI z-scores than non-carriers (P < 0.01), underscoring a clear genotype–phenotype correlation.

**Conclusion:**

This study broadens the catalog of genes associated with obesity in the Saudi population and demonstrates the value of studying extreme phenotypes in consanguineous families. Both previously recognized and novel variants were identified in association with severe, early-onset obesity, reflecting genetic heterogeneity across families rather than a single underlying mechanism. These findings provide a foundation for improved genetic characterization, tailored counseling, and future functional studies.

## Introduction

Obesity is one of the major causes of morbidity and mortality in developed countries and has increasingly spread in developing countries [1, 2]. Obesity is the accumulation of excess body fat due to a positive energy balance [3–5]. Over the past three decades, there has been a dramatic increase in the prevalence of individuals with obesity and overweight worldwide [6]. As is known, obesity has become a major epidemic and is not effectively controlled by therapeutic methods such as dietary and lifestyle modifications. According to previous studies, the number of individuals with obesity is expected to reach around 1.12 billion by 2030 [1].

Different factors contribute to the development of obesity, including increased intake of high-calorie foods and reduced physical activity [7]. Obesity can lead to several adverse effects, such as an increased risk of cardiovascular disease, diabetes, hypertension, arthritis, respiratory diseases, and cancer [8–10]. Among countries, Saudi Arabia has one of the highest prevalence rates of individuals with obesity and overweight, attributed to various environmental factors such as dietary behavior, mental health status, lifestyle, and lack of physical activity [5, 11–14]. Additionally, genetic mutations affecting hormones related to hunger, satiety, and energy metabolism regulation also play a role [1]. In Saudi Arabia, the proportion of individuals with obesity and overweight has significantly increased in recent years [15, 16].

Genetic predisposition plays a crucial role in the development of obesity syndromes in humans, including disorders that affect the endocrine nervous system, hormones regulating hunger and satiety, and imbalances or loss of function in hormone receptors [1]. Obesity results from a complex interplay of physiological systems, with the brain acting as the primary regulator. Several organ systems contribute to the regulation of energy. Circulating ‘adiposity signals,’ reflecting fat-mass size, integrate with satiety cues from the gastrointestinal tract to maintain energy balance [17].

Several hormones and genes previously described as contributing to and related to obesity include ghrelin (GHRL), leptin (LEP) [18], peptide YY, neuropeptide Y (NPY) [19], agouti-related protein (AGRP) neurons, and pro-opiomelanocortin (POMC), as well as cocaine- and amphetamine-related transcript (CART) neurons [20]. The hereditary aspects of obesity have long been recognized. However, the genetic contribution to this relatively rapid, population-wide change is becoming increasingly apparent. Recent advances in genotyping have enabled detailed exploration of obesity genetics, spanning single-gene defects that drive rare syndromes to the multiple loci underlying more common forms of the condition [21].

According to recent reports, more than 100 sites in the human genome have been identified as associated with obesity based on genome-wide association study (GWAS) findings [22]. Mutations in genes encoding enzymes and hormones involved in metabolic processes, appetite regulation, and maintenance of vital bodily functions are responsible for disorders associated with Mendelian forms of obesity, in which the phenotype is apparent [23].

Three different types of gene-level changes inevitably lead to genetic mutations. The first involves syndromic forms of obesity, characterized by features such as intellectual disability, male hypogonadism, extreme hunger, delayed growth, physical deformities, and obesity. At least 20 such syndromes have been discovered, including Bardet-Biedl syndrome (BBS), Cohen syndrome (COH), Prader-Willi syndrome (PWS), Alström syndrome (ALMS1), Fragile X syndrome, Borjeson-Forssman-Lehmann syndrome (BFLS), 16p11.2 deletion syndrome, Albright hereditary osteodystrophy (AHO), Kinase Suppressor of Ras 2 (KSR2) variants, and TUB mutations [24–36].

Monogenic obesity refers to rare forms of obesity resulting from mutations with large effect sizes in single or chromosomal genes [37]. Rare cases of monogenic obesity provided the first direct evidence that genetic factors play a substantial role in body weight regulation. Typically, a single gene mutation causes monogenic obesity, with conditions presenting in early childhood, often severely [38]. Some monogenes influence obesity by regulating metabolism, energy balance, appetite, and brain receptor function. Genetic defects at these sites disrupt their function or reduce their efficiency, such as imbalances in the hormone leptin (LEPH), secreted by lipid cells that regulate hunger and satiety, or defects in the leptin receptor (LEPR) and MC4R [39].

Polygenic obesity results from the combined action of multiple alleles from multiple genes, together with environmental risk factors [2]. Among the most prominent polygenes are mutations in the fat mass and obesity-associated gene (FTO) and defects in the insulin-induced gene 2 (INSIG2), both of which contribute to increased body fat and weight [40]. Recent advances in genome-wide association studies (GWAS) and large-scale multi-ancestry analyses have identified hundreds of loci associated with obesity, highlighting its complex genetic architecture [41]. Despite these advances, a substantial proportion of heritability remains unexplained (missing heritability), likely due to rare variants, gene–gene interactions, and environmental influences [42].

Given the sequential structure of the Saudi population, this study was undertaken. The aim of this study is to investigate genetic variants associated with obesity in consanguineous Saudi families and to identify potential candidate genes contributing to severe obesity.

## Results

### Manual analysis results

After performing manual filtration analysis for one individual with obesity and one control individual from each family, a list of 14 candidate genes was identified within these families (Tables 1 and 2). PCR sequencing analysis was conducted on seven of these genes. A set of forward (FW) and reverse (RV) primers was designed for each candidate gene to cover all exons. All results were negative, and no mutations were found in the list of genes screened by PCR analysis: ***TPK1, ID3, CLMP, SOD2, LDHAL6A, SENP1, DACH1.*** In silico prediction scores were available only for a subset of variants; unavailable values are indicated as (NA).

**Table 1:** Candidate genes identified after filtration and prioritization.

Family	Gene	Zygosity	Read depth	Population database (1000G/dbSNP)	In silico prediction
Family 1	*TPK1*	Homozygous	10	Not found	NA
Family 2	*ID3*	Homozygous	10	Not found	NA
Family 2	*CLMP*	Homozygous	27	Not found	NA
Family 3	*SOD2*	Homozygous	20	Not found	NA
Family 3	*LDHAL6A*	Homozygous	57	Not found	SIFT: 0.284; PolyPhen: 0.0
Family 3	*SENP1*	Homozygous	10	Not found	NA
Family 4	*SLC20A1*	Homozygous	11	Not found	NA
Family 4	*MLYCD*	Homozygous	16	Not found	NA
Family 4	*ACSF3*	Homozygous	48	Not found	NA
Family 5	*DACH1*	Homozygous	45	Not found	NA
Family 6	*FGFRL1*	Homozygous	23	Not found	NA
Family 6	*ABCB5*	Homozygous	19	Not found	SIFT: 0.0; PolyPhen: 1.0
Family 7	*NBEA*	Homozygous	26	Not found	NA
Family 7	*METRNL*	Homozygous	93	Not found	NA
Family 8	No candidate genes identified after filtration	NA	NA	NA	NA

NA: not available. The population database refers to the absence of variants in the 1000 Genomes Project and dbSNP, as indicated by the filtration output. In silico predictions are reported where available using SIFT and PolyPhen-2 scores. Zygosity indicates the genotype of the identified variant after filtration.

**Table 2:** Results of rare genes from diploid (moon) after sanger sequencing analysis.

**12- Family** **13-** **14-**	**11- The number of family members**	**10- Case used in WES**	**9- Parents**	**8- Number of individuals had gene mutation**	**7- Name of the genes**	**6- Zygosity**	**Effect**	**Variant**	**Variant effect protein**	**5- Symptoms at birth of individuals**	**3- Artificial intelligence report** **4- (Diploid Moon)**	**2- Database report**
24- Family 1	23- 6	22- Girl, 25-year-old with BMI 47.5 kg/m²	20- The father is a heterozygous mutation. 21- The mother is deceased.	19- 2	18- ***ALMS1***	Hetro	Missense	T>C	Protein change: Not specified (transcript-dependent)	17- Normal weight at birth but started to gain weight rapidly, have severe early-onset obesity and hyperphagia.	16- Novel and prediction polymorphism.	15- Novel
				28- 5	27- ***BBS10***	Hetro	Frameshift	DelA AGA	p.Lys243fs		26- Previously described and prediction disease.	25- Previously described
				32- 4	31- ***MAGEL2***	Hetro	Missense	G>T	p.Val225Leu		30- Novel and prediction polymorphism.	29- Novel
				36- 4	35- ***ABCB4***	Hetro	Missense	C>T	p.Thr34Met		34- Previously described and prediction polymorphism.	33- Previously described
				40- 4	***39***- ***VPS13B***	Hetro	Missense	T>C	p.Leu2868Ser		38- Novel and prediction disease.	37- Novel
49- Family 2 50-	48-	47- Girl, 11-year-old with BMI 30.8 kg/m²	45- The father is a heterozygous mutation. 46- The mother is lean.	44- 4	***PCNT***	Hetro	Missense	C>A	p.Thr78Asn	43- Normal weight at birth but started to gain weight rapidly, have severe early-onset obesity and hyperphagia.	42- Novel and prediction polymorphism.	41- Novel
				54- 4	*53- **RAI1***	Hetro	Missense	A>C	p.Lys1003Thr		52- Novel and prediction polymorphism	51- Previously described
				58- 4	*57*- ***CIDEC***	Hetro	Missense	G>C	p.Val653Leu		56- Novel and prediction disease.	55- Novel
				62- 4	***61- TTC8***	Hetro	Missense	G>A	p.Arg192His		60- Novel and prediction disease.	59- Previously described
				66- 2	***65- MAGEL2***	Hetro	Missense	G>A	p.Met125Ile		64- Novel and prediction polymorphism.	63- Novel
88- Family 3 89-	87- 4	86- Boy, 8-year-old with BMI 33 kg/m².	84- The father is a heterozygous mutation. 85- The mother is lean.	83- 4	***H6PD***	Hetro	Missense	c.1747G>C	p.Glu583Gln	82- Normal weight at birth but started to gain weight rapidly, have severe early-onset obesity and hyperphagia.	81- Novel and prediction disease.	80- Novel
				93- 5	***92- SPG11*** Exon 35	Hetro	Splice region	G>T	p.Pro2195Pro		91- Novel and prediction disease.	90- Previously described
				97- 5	96- ***SPG11*** Exon 39	Hetro	Missense	T>C	p.Phe2378Leu		95- Previously described prediction disease.	94- Novel
				101-5	100- ***CIDEC***	Hetro	Missense	G>A	p.Arg192His		99- Novel and prediction disease.	98- Novel
109- Family 4 110-	108- 6	107- Girl, 15-year-old with BMI 30 kg/m².	106- The mother is a heterozygous mutation. The father is lean.	105- 3	***SETD2***	Hetro	Missense	A>G	p.Tyr428Cys	104- Normal weight at birth but started to gain weight rapidly, have severe early-onset obesity and hyperphagia.	103- Novel and prediction disease.	102- Novel
				114- 4	113- ***EHMT1***	Hetro	Missense	G>A	p.Arg387His		112- Previously described and prediction polymorphism.	111- Previously described
				118- 3	117- ***ALMS1***	Two Hetro	Frameshift	DelGG	p.Glu20fs		116- Novel and prediction disease.	115- Novel
							Frameshift	DelG	p.Glu21fs			
				122- 4	121- ***MCM3AP***	Hetro	Missense	G>A	p.Arg1179His		120- Novel and prediction polymorphism.	119- Novel
				126- 5	125- ***MKS1***	Hetro	Missense& spliceRegion	A>G	p.Ser64Gly		124- Novel and prediction disease.	Novel
149- Family 5	147- 7 148-	146- Boy, 16-year-old with BMI 33.4 kg/m².	144 The father is a heterozygous mutation 145- The mother is lean.	143- 3	***MC4R***	Hetro	Missense	C>T	p.Thr162Ile	142- Normal weight at birth but started to gain weight rapidly, have severe early-onset obesity and hyperphagia	141- Previously described and prediction disease.	140- Previously described
				153- 4	152- ***ALMS1***	Two Hetro	Missense	A>G	p.Tyr1012Cys		151- Novel and prediction polymorphism.	150- Novel
							Missense	A>G	p.Lys3520Arg			
162- Family 6	160- 7 161-	159- Girl, 7-year-old with BMI 23 kg/m².	157- The father is a heterozygous mutation. 158- The mother is lean.	156- 2	***DYRK1B***	Hetro	Missense	C>A	p.Gln585Lys		155- Novel and prediction disease.	154- Novel
				166- 5	165- ***IGF1R***	Two Hetro	Missense	G>A	p.Arg1357Gln		164- Novel and prediction disease	163- Novel
							Missense	G>A	p.Ala1338Thr		168- Previously described and prediction disease.	167- Previously described
178- Family 7	176- 6 177-	175- Girl, 18-year-old with BMI 43 kg/m².	172- The mother is a heterozygous 173- mutations. 174- The father is lean.	171- 4	***TRAPPC9***	Hetro	Missense	G>A	p.Arg145Gln		170- Previously described and prediction disease.	169- Previously described
Family 8	^*^There is no candidate genes with obesity											

### Diagnosis of rare variant using diploid (moon) software

A report on variants was generated, yielding a list of genes associated with the observed phenotypes. The reports provided a list of rare genes and their classification for each family. In total, 35 genes and 40 variants were initially identified across all families. Among these, 19 candidate genes encompassing 28 variants were prioritized based on their functional relevance to obesity and supporting evidence from previous studies.

The list of rare genes and variants was compiled from Diploid (Moon) software and included the following candidate genes associated with obesity: ***ALMS1, BBS10*, *BLK, PDSS1, MAGEL2, ABCB4, VPS13B***, ***PCNT, RAI1, CIDEC, TTC8, MAGEL2, C15orf38-AP3S2, H6PD, SPG11, CIDEC, CEP164, SETD2, EHMT1, ALMS1, MCM3AP, MKS1, PRMT7*** and ***KMT2D.*** Candidate genes were selected based on their known or plausible functional relevance to obesity, including roles in metabolic pathways, adipose tissue regulation, or previously reported associations with obesity-related phenotypes. PCR sequencing analysis was performed on the 19 candidate genes. For each gene, a set of forward (FW) and reverse (RV) primers was designed to cover specific exonic regions. Sanger sequencing confirmed the presence of variants in these genes, resulting in the identification of 27 variants across 19 genes, including ***ALMS1, BBS10, MAGEL2, ABCB4, VPS13B, PCNT, RAI1, CIDEC, TTC8, H6PD, SPG11, SETD2, EHMT1, MCM3AP, MKS1, MC4R, DYRK1B, IGF1R*** and ***TRAPPC.*** The discrepancy between manual filtration and Diploid (Moon)-based results likely reflects differences in variant prioritization strategies, where the integrative annotation framework of Moon enabled the identification of additional candidate variants. The reported ALMS1 variant was reviewed in the context of transcript variability, and amino acid annotation may differ depending on the reference transcript used.

## Discussion

Our findings highlight the contribution of genetic variation to severe obesity in this population, particularly within consanguineous families. This could help explain the increased prevalence of obesity in Saudi society and contribute to exploring the genetic factors involved in the progression of obesity. The study aims to determine monogenic mutations responsible for obesity among Saudi children with a BMI ≥ 30 kg/m^2^. Monogenic obesity refers to several rare forms of severe obesity caused by mutations with a major effect on a single gene. Despite their rarity, studies of monogenic obesity have greatly contributed to understanding the pathogenesis of obesity and the pathways and mechanisms involved in its development [1, 43, 44].

Globally, monogenic forms of obesity have been demonstrated in approximately five percent of individuals with the most severe forms of obesity [1]. This study describes an in-depth analysis of eight consanguineous Saudi families with individuals presenting extreme obesity, using mature technologies for variant discovery (WES and Sanger sequencing). The study included eight families (Families 1–8). The individual with the highest BMI in each family was selected for WES. After filtering and prioritizing WES variants through manual filtration, a list of candidate genes, *TPK1, ID3, CLMP, SOD2, LDHAL6A, SENP1,* and *DACH1*, was developed. Although the candidate genes showed high coverage, sequencing revealed no positive results. Similar studies have reported similar findings [45, 46], which have been attributed to uneven enrichment in certain genomic regions [47].

The absence of validated variants following manual filtration may reflect limitations in filtering strategies and the complexity of variant prioritization in small cohorts. The use of novel bioinformatic software is therefore essential. Diploid (Moon) is a software platform that uses artificial intelligence techniques to analyze genomic data. Utilizing this software yielded interesting results in our study, revealing heterozygous mutations in several genes of significant interest, including ALMS***1**, **CIDEC***, ***SETD2***, ***MC4R***, and ***DYRK1B***. Several of the identified genes, including well-established obesity-related genes such as MC4R and ALMS1, have documented links to obesity, whereas others may represent candidate genes with varying levels of supporting evidence. Particularly, ***MC4R*** is widely recognized as one of the leading genetic contributors to obesity worldwide [33, 48, 49]. In this study, most of the identified genetic mutations were heterozygous. The predominance of heterozygous variants observed in this study may reflect genetic heterogeneity and the contribution of different inheritance patterns, including dominant or incompletely penetrant variants, rather than strictly autosomal recessive models. While consanguinity in these families would typically suggest a recessive inheritance model, the predominance of heterozygous variants indicates a more complex genetic architecture. This may reflect genetic heterogeneity across families, variable inheritance patterns, or limitations in variant detection and prioritization. Therefore, these findings should be interpreted with caution and do not necessarily indicate a polygenic effect within individual cases. These findings further support the role of genetic heterogeneity in obesity, with different candidate variants identified across families [23, 50–53]. Reviewing various genetic studies conducted in Saudi Arabia, to our knowledge, several of the candidate genes identified in this study have not been widely reported in Saudi families with obesity. Each of these genes has a distinct impact on the development of obesity.

The ***ALMS1*** gene, which is highly expressed in the gastrointestinal tract, has demonstrated a strong phenotype in all reported individuals carrying a mutation in this gene [26]. Regarding the ***SETD2*** gene, it is associated with overgrowth syndrome, where most individuals carrying mutations present with severe obesity. Highly expressed adipose tissue genes were also identified in this study [54].

The ***DYRK1B*** gene is highly expressed not only in adipose tissue but also in the gastrointestinal tract, endocrine tissues, the cerebral cortex, bone marrow, and the immune system. Mutations in ***DYRK1B*** have been linked to abdominal obesity–metabolic syndrome 3, an autosomal dominant condition [49]. The ***VPS13B*** gene is also highly expressed in adipose tissue [33]. In this study, a homozygous mutation in ***VPS13B*** was identified in one individual. VPS13B is classically associated with Cohen syndrome, an autosomal recessive disorder in which truncal obesity may develop; therefore, biallelic or homozygous variants are more consistent with a syndromic obesity phenotype than isolated obesity. This individual exhibited extremely high BMI scores, supporting a possible link between VPS13B-related phenotypes and severe obesity. 

These findings further support the role of genetic heterogeneity in obesity, with different candidate variants identified across families. The different results observed in this study collectively highlight a significant genetic contribution to obesity. This study should be interpreted in light of several methodological considerations. Homozygosity mapping was not incorporated into the analytical pipeline, although it could have further refined candidate regions in this consanguineous cohort. In addition, the analysis relied primarily on a single variant-prioritization platform (Moon), and no functional validation was performed to confirm the pathogenicity of the identified variants, which limits the strength of causal inferences. Future studies integrating complementary bioinformatics approaches and experimental validation may enhance variant prioritization and improve the robustness of the findings. Furthermore, the use of age- and sex-specific pediatric BMI classifications, along with recruitment through pediatric referral, may introduce variability and potential selection bias, thereby limiting the generalizability of the results.

## Methods

### Study subjects

The study samples were collected at the Department of Genetics at King Faisal Specialist Hospital and Research Center, which is part of the Saudi Human Genome Project. The ethics committee approved the study at the Obesity Chair, School of Medicine, King Saud University (RAC number 2080036). Samples were collected from eight Saudi families, each comprising 6 to 9 members, totaling 56 individuals, including healthy-weight and obese individuals (both adults and children) aged 2 to 30 years (Table 1). In each family, one parent was considered a putative carrier based on family segregation patterns and the presence of heterozygous variants identified during genetic analysis, while one or more children were affected. Samples from individuals with obesity were compared with those from their healthy siblings who had neither developed obesity nor were carriers of the condition (controls). Samples were collected from families affected by obesity across different regions in the Kingdom of Saudi Arabia. Consanguineous families were selected from four cities: Riyadh, Jeddah, Makkah, and Jazan. Each family included at least three affected individuals with severe early-onset obesity. A total of 30 adults and children were identified with obesity or severe obesity from different families. For adults, obesity and severe obesity were defined as a BMI ≥ 30 kg/m^2^. For children, classifications were based on a BMI above the 90th percentile (overweight) and the 97th percentile (obesity), respectively. Families were identified with the assistance of local pediatricians, and written informed consent was obtained from the children’s parents or legal guardians. For WES, one affected child and one unaffected control child were selected from each family. The affected child was selected as the sibling with the highest BMI within the family. The control child was selected from a non-obese sibling within the same family, preferably of comparable age and sex when available, and with no clinical evidence of obesity at the time of recruitment.

### Clinical characterization

The families were assessed to collect information on the children’s prenatal, perinatal, and neonatal medical history. A detailed pedigree was constructed, and the genealogical relationships within the families were cross-checked after interviewing family members and measuring individuals’ heights (cm), waist circumferences (cm), and weights (kg). Blood samples were collected from each individual in these families, and DNA extraction was performed using Gentra Puregene Blood Kits (Qiagen, Crawley, UK). The study was conducted in the Research Center (Genetics Department) at King Faisal Specialist Hospital in Riyadh (Fig. 1 and Table 3). Waist circumference was recorded but not included in the final analysis due to incomplete data across participants.

**Figure 1:**
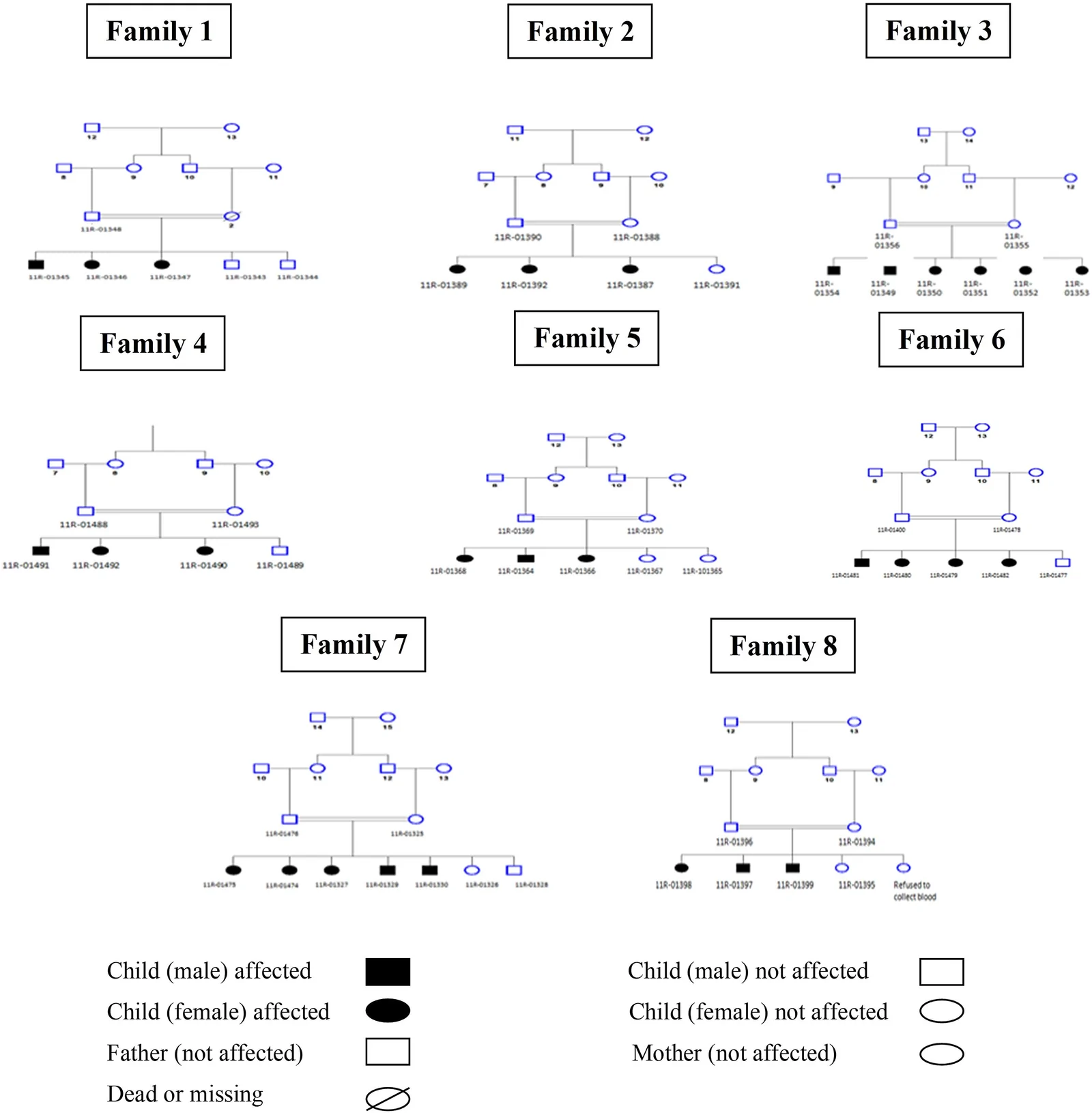
Pedigrees of eight Saudi families with individuals affected by or carrying monogenic forms of obesity. These pedigrees illustrate an autosomal recessive inheritance pattern. Affected individuals are shaded in black. Parents and individuals with a healthy weight from the same families are shown in clear color.

**Figure 2:**
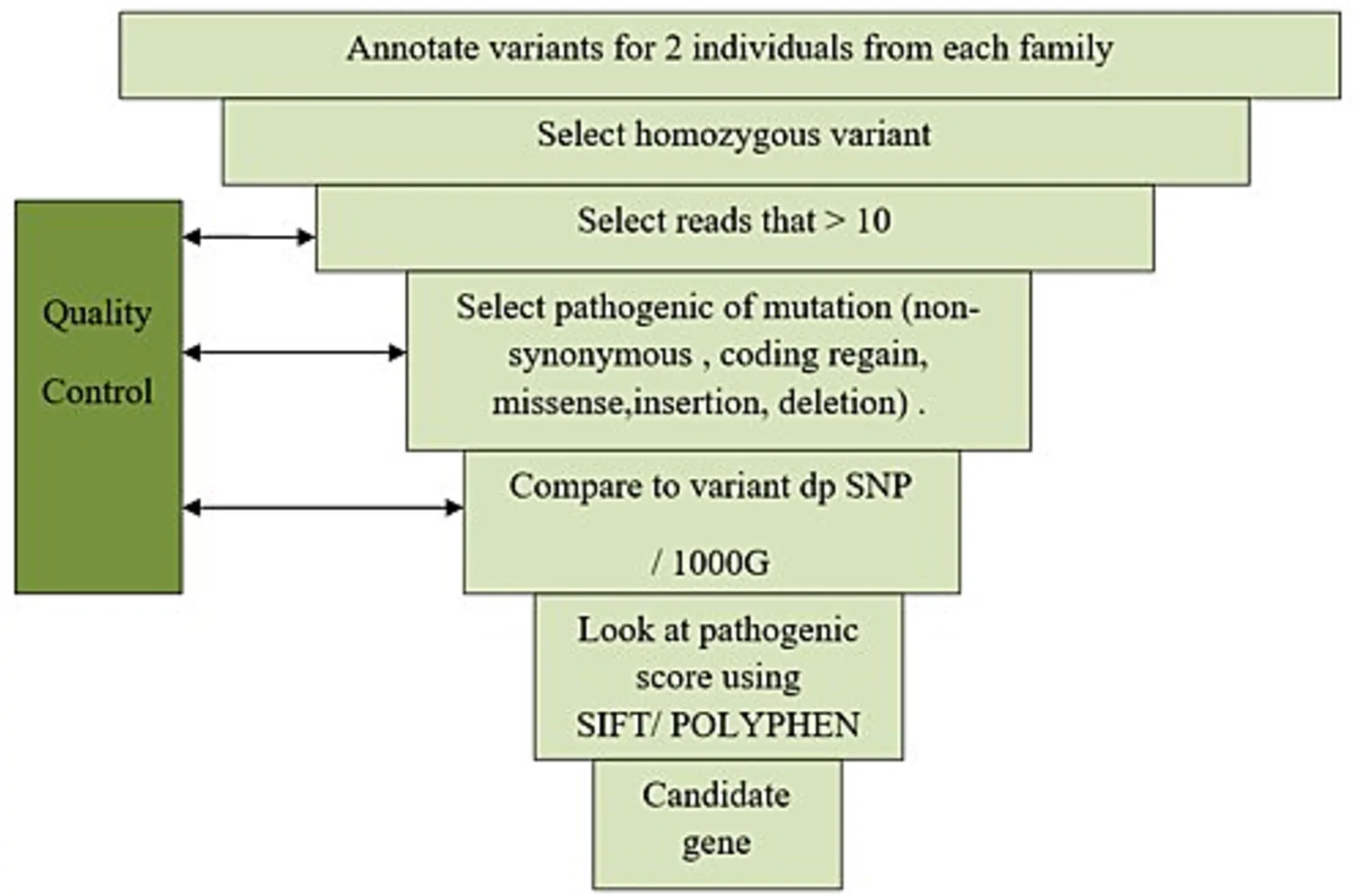
Steps of filtration of the VCF file.

**Table 3:** Phenotypic characteristics of the study families.

**Family**	**Relationship**	**Age (years)**	**Sex**	**Obesity status**	**Adult BMI (kg/m** ^**2**^ **)**	**Pediatric BMI growth-chart equivalent**
Family 1	Son	18	M	OB	NA	32.3
Family 1	Son	13	M	OB	NA	31.4
Family 1	Son	16	M	OB	NA	30.0
Family 1	Daughter	25	F	OB	47.5	NA
Family 1	Daughter	30	F	OB	65.0	NA
Family 1	Father	NA	M	OB	30.1	NA
Family 1	Mother	Deceased	NA	NA	NA	NA
Family 2	Father	NA	M	OB	30.0	NA
Family 2	Mother	NA	F	Normal	20.0	NA
Family 2	Daughter	17	F	OB	NA	25.0
Family 2	Daughter	16	F	OB	NA	31.0
Family 2	Daughter	11	F	OB	NA	30.8
Family 2	Daughter	8	F	OB	NA	23.0
Family 3	Son	20	M	OB	62.0	NA
Family 3	Daughter	13	F	OB	NA	30.1
Family 3	Daughter	21	F	OB	32.0	NA
Family 3	Daughter	16	F	OB	NA	33.0
Family 3	Daughter	18	F	OB	NA	34.0
Family 3	Son	8	M	OB	NA	33.0
Family 3	Mother	NA	F	Normal	22.0	NA
Family 3	Father	NA	M	Normal	23.0	NA
Family 4	Father	NA	M	OB	30.5	NA
Family 4	Mother	NA	F	Normal	23.2	NA
Family 4	Son	17	M	OB	NA	30.1
Family 4	Daughter	15	F	OB	NA	30.0
Family 4	Son	10	M	OB	NA	22.6
Family 4	Daughter	2	F	OB	NA	20.0
Family 5	Son	13	M	OB	NA	33.0
Family 5	Daughter	12	F	OB	NA	26.0
Family 5	Daughter	18	F	OB	NA	32.2
Family 5	Daughter	8	F	OB	NA	22.0
Family 5	Daughter	16	F	OB	NA	33.4
Family 5	Father	NA	M	Normal	27.0	NA
Family 5	Mother	NA	F	Normal	28.0	NA
Family 6	Son	12	M	Normal	NA	16.8
Family 6	Father	NA	M	Normal	NA	NA
Family 6	Son	8	M	OB	NA	20.8
Family 6	Daughter	10	F	OB	NA	23.0
Family 6	Daughter	11	F	OB	NA	24.0
Family 6	Daughter	7	F	OB	NA	23.0
Family 6	Mother	NA	F	OB	35.0	NA
Family 7	Mother	NA	F	Normal	48.5 before surgery	NA
Family 7	Daughter	9	F	Normal	NA	21.0
Family 7	Daughter	11	F	OB	NA	29.0
Family 7	Son	10	M	Normal	NA	25.0
Family 7	Son	19	M	OB	38.0	NA
Family 7	Son	17	M	OB	NA	39.0
Family 7	Daughter	18	F	OB	NA	43.0
Family 7	Father	NA	M	Normal	27.7	NA
Family 7	Daughter	20	F	OB	40.0	NA
Family 8	Father	NA	M	Normal	33.4	NA
Family 8	Mother	NA	F	Normal	34.5	NA
Family 8	Daughter	21	F	OB	36.0	NA
Family 8	Daughter	NA	F	Normal	24.0	NA
Family 8	Son	9	M	OB	NA	21.0
Family 8	Son	17	M	OB	NA	33.0

Abbreviations: OB, obesity; BMI, body mass index; NA, not available or not applicable. Adult BMI was reported for adult participants, whereas pediatric BMI values were interpreted according to age- and sex-specific growth-chart classifications.

### DNA extraction

Blood samples were collected in EDTA tubes for DNA extraction and stored at 4°C to −80°C until required. Approximately 3 mL of blood was collected per 15 mL tube. DNA concentration, quantification, and purity were measured using the NanoDrop spectrophotometer, and the stock DNA solution was diluted before the experiments.

### Whole exome sequencing (WES)

Whole Exome Sequencing (WES) was performed on samples collected from two children in each family, including one child with obesity and one control child without obesity, by the sequencing core facility at the Department of Genetics, Research Center, King Faisal Specialist Hospital and Research Center (KFSHRC) in Riyadh. The child with the highest BMI was selected for WES among affected individuals in each family. Sequencing was performed using a validated exome capture protocol following the standard workflow of the sequencing core facility at the Department of Genetics, KFSHRC, including library preparation, exome capture, sequencing, and primary variant calling. Candidate variants were subsequently validated by PCR amplification and Sanger sequencing. Sequence data were analyzed and interpreted using SeqMan software to confirm variant calls and assess sequence quality.

### WES filtration protocol

Filtering was performed to simplify the annotation process, identifying whether variants were homozygous or heterozygous, their genomic locations, associated gene symbols, and whether they were exonic, splice-site (donor or receptor), intronic, or intergenic, including transcription factors. All variants generated from Genome Quest were saved on the company’s servers. Genome Quest, a sequencing data management company based in the US (http://www.genomequest.com), has a contract with the Genetics Department at King Faisal Specialist Hospital and Research Center to perform bioinformatics work and generate variant calls from Illumina MiSeq and Qual files. Genome Quest applied quality control measures, mapped sequencing reads to the reference genome and conducted variant calling. Variants were filtered using standard quality-control criteria, population-allele-frequency thresholds, and predicted functional impact.

### Data analysis

This study used two bioinformatics methods to identify candidate genes associated with obesity: manual filtering of VCF files and the Diploid (Moon) software for rare-variant diagnosis. Diploid is a world-class software platform that provides comprehensive clinical diagnostics and precision medicine services for rare disorders. Using artificial intelligence, the platform converts VCF files into a medical report, signed by a board-certified medical geneticist (MD). Phenotypic data are entered manually, and the platform automatically compiles a list of genes associated with the reported symptoms. Among tens of thousands of variants, the program retains about 1–3 variants most likely to be responsible for the individual’s phenotype (Fig. 1).

The manual prioritization process included assessments of variant type, inheritance pattern, allele frequency, predicted functional impact, and gene-phenotype relevance. Functional prediction was evaluated using in-silico tools, including SIFT, PolyPhen-2, and MutationTaster. Variant interpretation considered available evidence, including population frequency, predicted consequence, segregation pattern, prior reports, and phenotype relevance, in line with general American College of Medical Genetics and Genomics/Association for Molecular Pathology principles where applicable. For phenotypic analysis, descriptive statistics were calculated for the study cohort and stratified according to affected and unaffected status. Continuous variables, including age and BMI-related measures, were summarized as mean ± standard deviation and range, while categorical variables were summarized as frequencies and percentages. BMI z-scores were compared between carriers and non-carriers using an appropriate statistical test based on data distribution and sample size. The test statistic, P value, and effect size are reported where applicable. A P value < 0.05 was considered statistically significant. The term (BMI equivalent in Growth Chart) refers to pediatric BMI classification derived from age- and sex-specific growth charts.

### Primer design

Primers were designed using Primer3 software. Reference genomic sequences were retrieved from genome browsers, including UCSC Genome Browser and Ensembl, and primer specificity and suitability were checked using OLIGO and related primer evaluation tools. The candidate genes and families in which they were sequenced 26 genes (***TPK1***, ***ALMS1***, ***BBS1***, ***MAGEL2***, ***ABCB4***, ***VPS13B***, ***ID3***, ***CLMP***, ***PCNT***, ***RAI1***, ***CIDEC***, ***TTC8***, ***SOD2***, ***LDHAL6A***, ***SENP1***, ***H6PD***, ***SPG11***, ***SETD2***, ***EHMT1***, ***MCM3AP***, ***MKS1***, ***DACH1***, ***MC4R***, ***IGF1R***, ***DYRK1B***, ***TRAPPC9***).

### Polymerase chain reaction (PCR)

The name of the kit used in the reaction was (GC-Hotstart (F) DNA polymerase kit,100RX, Cat. No. GC-100, Store@amblent, let No. DB300602). The Thermocycler used was an Applied Biosystems Veriti 96-Well Programmable Thermocycler. The discrepancy between manual filtration and Diploid (Moon)-based results may reflect differences in variant prioritization strategies, where manual filtration yielded no validated variants, while the integrative approach of Moon identified additional candidate variants for validation.

### Sanger sequencing

Sanger sequencing was used to validate variants caused by mutations in candidate genes. We identified all exons and are using the SEQMAN software to read the gene and identify mutations. Variants were validated by comparing affected and unaffected family members where DNA was available, and segregation with the obesity phenotype was assessed within each family when possible. Representative Sanger sequencing traces are provided (Supplementary Fig. S1–S3) to demonstrate variant validation and sequence quality.

### Pathogenicity assessment

Pathogenicity was assessed using both manual curation and in silico prediction tools. Manual curation included assessment of codon change, predicted amino acid substitution, variant type, gene function, inheritance pattern, and consistency with the observed phenotype. In silico prediction tools included SIFT, PolyPhen-2, and MutationTaster.

The outputs of these tools were interpreted collectively rather than independently, with greater priority given to variants consistently predicted to be damaging or deleterious across multiple tools. Variant classification was further guided, where applicable, by ACMG/AMP criteria, including population frequency, computational evidence, predicted functional consequence, segregation evidence, and previously reported disease associations. Variants were classified as pathogenic, likely pathogenic, variant of uncertain significance, likely benign, or benign based on available evidence. Variants were annotated with respect to a reference transcript to ensure consistency in variant reporting. In addition, the analytical workflow was clarified to distinguish between the two approaches used: manual filtering and Diploid (Moon) based prioritization. Manual filtering was based on variant quality, allele frequency, predicted functional impact, and gene–phenotype relevance, whereas Diploid (Moon) analysis incorporated phenotype-driven prioritization, inheritance modeling, and integrated variant annotation to rank candidate variants. BMI z-scores were compared between carriers and non-carriers using an appropriate statistical test based on data distribution and sample size, as specified in the revised Methods section. Results are reported with the test statistic, P value, effect size, and 95% confidence interval where applicable. A P value < 0.05 was considered statistically significant.

## Conclusion

The factors responsible for the spread of obesity over the past three decades include increased intake of high-calorie foods and reduced physical activity. Saudi Arabia is now among the nations with the highest prevalence rates of individuals with obesity and overweight, attributed to multiple predisposing environmental factors such as dietary behavior, mental health status, lifestyle, and lack of physical activity, in addition to genetic mutations in hormones related to hunger, satiety, and the regulation of energy metabolism. Using the latest analytical techniques, we have demonstrated the genetic contribution to obesity in the Saudi population. Interestingly, the findings suggest genetic heterogeneity among the studied families, with distinct candidate variants identified within each family. However, the current study design does not allow definitive conclusions regarding inheritance patterns or causal relationships within individual cases. Most of the genes identified in this study are highly expressed in adipose tissue, the brain, and the gastrointestinal tract.

## Supplementary Material

Supplementary_Sanger_Traces_ddag054

## Data Availability

All data generated or analyzed during this study are available in the paper and its Supplementary Information section. Further inquiries can be directed to the corresponding author.
