# Supplementary material for: The monogenic forms of obesity in Saudi families
Source: Hum Mol Genet. 2026 Oct 19;35(21):ddag054. doi: 10.1093/hmg/ddag054 (PMC13679745; doi:10.1093/hmg/ddag054)
Supplement: Supplementary file 1 [file supplementary_sanger_traces_ddag054.docx]

Supplementary Figure S1–S3: Sanger Sequencing Traces

Figure S1. Representative Sanger chromatogram for TPK1 (Family 1). The arrow indicates the variant position.

Figure S2. Representative Sanger chromatogram for MC4R showing a heterozygous variant .

Figure S3. Representative Sanger chromatogram for SETD2 variant.
